# How Sponges Are Caught

**Published:** 1881-11

**Authors:** 


### HOW SPONGES ARE CAUGHT.

A correspondent of the New Haven (Conn.) *Register* tells how they fish for sponges in the Bahamas : When a vessel arrives at the fishing ground it is anchored, and the men in small boats proceed to look for sponges in the water below. The water is a beautiful light blue color, and so clear a sixpence can easily be seen on the white, sandy bottom in thirty-five to forty feet of water. Of course when there is no wind, and the surface of the water is still, the sponges are easily seen, but when a gentle breeze is blowing a "sea-glass" is used. A sea-glass consists of a square pine box about twenty inches in length, a pane of glass about 10x12 inches placed in one end, water-tight. To use it, the glass end is thrust into the water, and the face of the operator is placed close to the other. By this means the wave motions of the water are overcome, and the bottom readily seen. Sponges when seen on the bottom attached to rocks, look like a big black bunch. They are pulled off their natural beds by forked hooks, which are run down under the sponge, which is formed like the head of a cabbage, and the roots pulled from the rocks.

When brought to the surface it is a mass of soft, glutinous stuff, which to touch feels like soap or thick jelly. When a small boat-load is obtained they are taken to the shore, where a crawl is built, in which they are placed to die, so that the jelly substance will readily separate from the firm fibre of the sponge. These crawls are built by sticking pieces of brush into the sand out of the water, large enough to contain the catch. It takes from five to six days for the insect to die, when the sponges are beaten with small sticks, and the black glutinous substance falls off, leaving the sponge, after a thorough washing, ready for market. To the fishermen generally the occupation is not a lucrative one. I am told the wages will hardly average three dollars per week, beside board. There is but little diving for sponges, except for a particularly fine bunch, which cannot be got with the hook. The sponge is formed by small insects, and is the hive in which they live. Different qualities are found growing side by side, although in certain regions the finer and more valuable sponges are found.

---

Dogs in a state of nature never bark ; they whine or growl. The explosive noise is only found among those which have been domesticated.

